# Genomic surveillance of the pneumonia outbreak caused by *Mycoplasma pneumoniae* among patients in Russia from January 2024 to June 2025

**DOI:** 10.3389/fcimb.2026.1736929

**Published:** 2026-03-25

**Authors:** Elena V. Korneenko, Irina S. Rog, Ivan K. Chudinov, Anfisa I. Kozyreva, Daria A. Sandanova, Alexander V. Pavlenko, Daria S. Matyushkina, Vadim M. Govorun

**Affiliations:** 1 Laboratory of Multiomics Research, Scientific Research Institute for Systems Biology and Medicine, Federal Service on Consumer Rights Protection and Human Well-Being Surveillance, Moscow, Russia; 2 Moscow Institute of Physics and Technology National Research University, Moscow, Russia; 3 Laboratory of Mathematical Biology, Scientific Research Institute for Systems Biology and Medicine, Federal Service on Consumer Rights Protection and Human Well-Being Surveillance, Moscow, Russia; 4 Symple Systems Laboratory, Scientific Research Institute for Systems Biology and Medicine, Federal Service on Consumer Rights Protection and Human Well-Being Surveillance, Moscow, Russia

**Keywords:** *Mycoplasma pneumoniae*, co-infections, macrolide resistance, P1 adhesin, community-acquired pneumonia

## Abstract

**Background:**

*Mycoplasma pneumoniae* (MP) is one of the major pathogens that causes respiratory tract infections, including community-acquired pneumonia (CAP). The aim of the current study was to conduct molecular genetic surveillance of an outbreak of pneumonia caused by MP in various regions of the Russian Federation between January 2024 and June 2025.

**Methods:**

MP, viral and bacterial co-infections were detected in 482 nasopharyngeal swabs from patients with CAP using real-time PCR method. To investigate the mutations associated with resistance to macrolides and quinolones we describe the development and usage of primer panels for the complete *mgpA*, 23S, *parC*, *parE*, *gyrA*, *gyrB* genes followed by high-throughput sequencing. To support the results of PCR for MP we applied the ELISA for 75 serum samples.

**Results:**

MP was confirmed in 81.5% samples by real-time PCR and in 69.3% samples by ELISA. Bacterial co-infections were identified in 27.8% samples. *H. influenzae* was the most prevalent, detected in 19.7% samples, followed by *S. pneumoniae* (13.1%) and *C. pneumoniae* (0.8%). The most prevalent viral co-infections were RSV (16.2%), RV (14.7%), and hPIVs (9.5%). The A2063G mutation associated with macrolide resistance was found in 23% samples. Point mutations, A2064G and A2064C, were detected in 6 samples (1,8%). No significant mutations associated with resistance to quinolones were identified according to the sequencing of complete *parC*, *parE*, *gyrA*, *gyrB* genes. The phylogenetic analysis revealed that the *mgpA* gene sequences formed two distinct clades, 97.2% were classified as P1 type 1, while the remaining 2.8% were classified as P1 type 2.

**Conclusion:**

This study demonstrates a fundamental shift in the epidemiology of MP in the post-COVID-19 era, characterized by a transition from cyclical epidemics to year-round endemic circulation. We document increased disease severity, a dynamic profile of viral and bacterial co-infections, and significant geographic heterogeneity in macrolide resistance rates, which ranged from 0% to 50% across regions. The overall macrolide resistance rate was 23%, which is lower than previously reported. Furthermore, genotyping of the complete P1 adhesin gene revealed divergence, with a majority of sequences clustering within P1 type 1 and a minority within P1 type 2.

## 1 Introduction

*M. pneumoniae* (MP) is one of the smallest known cellular life forms, belonging to the class *Mollicutes*, which causes the infection of upper respiratory tract, including community-acquired pneumonia (CAP), pharyngitis, tracheitis, and tracheobronchitis (Meyer Sauteur et al., 2016). Infection caused by MP tend to be endemic, infecting of all age groups, but predominantly school-aged children (Waites and Talkington, 2004). Following the relaxation of the Coronavirus Disease 2019 (COVID-19) restrictions, a global outbreak of pneumonia caused by MP rapidly disseminated, originating in China and subsequently spreading throughout Asia and Europe (ESGMAC MAPS Study Group et al., 2025). Historically characterized by seasonal peaks in late autumn and winter, the epidemiology of pneumonia caused by MP (MPP) has shifted markedly in the post-COVID-19 era. While peak prevalence traditionally occurs in late autumn and winter, cases in the post-pandemic era have been detected year-round (Waites et al., 2017; Yan et al., 2024; Zhang et al., 2024).

In China during the 2023–2024 period over 1.6 million cases of acute respiratory infections were diagnosed year-round, with an MP infection rate of 35.43% (Sun et al., 2025). In the etiology of CAP outbreaks in the Russian Federation in 2024, MP was the predominant pathogen, identified in 89.4% of outbreaks. It was detected both as the single pathogen (64.2%) and in association with other microorganisms (25.2%) according to data provided by the Federal Service on Consumer Rights Protection and Human Well-Being Surveillance (available online https://www.rospotrebnadzor.ru/upload/iblock/b8a/u6lsxjabw032jkdf837nlaezxu3ue09m/GD_SEB.pdf).

MP acts not only as a primary causative agent of atypical pneumonia but also as a secondary pathogen. In immunocompromised individuals, MP can complicate pre-existing bacterial or viral infections, thereby worsening the disease course (Morris et al., 2017; Ozaras et al., 2020; Waites and Talkington, 2004). Common co-infecting pathogens include bacteria such as *Streptococcus pneumoniae*, *Chlamydophila pneumoniae*, *Haemophilus influenzae*, *Staphylococcus aureus* and *Klebsiella pneumoniae*, as well as viruses such as rhinovirus, adenovirus, parainfluenza viruses, respiratory syncytial virus, bocavirus, and SARS-CoV-2 (Li et al., 2022; Liu et al., 2023; Song et al., 2015; Teng et al., 2019; Zhang et al., 2018; Zhao et al., 2019; Zhou et al., 2020).

The clinical course of MP infection is characterized by a gradual onset, a persistent, dominant cough, and significant discrepancies between mild clinical symptoms and radiographic findings (Kutty et al., 2024; Waites et al., 2017). A key hallmark of the disease is its association with a diverse extrapulmonary manifestations, which range from cutaneous eruptions such as Stevens-Johnson syndrome to severe neurological complications such as encephalitis (Atkinson et al., 2008; Meyer Sauteur, 2024; Waites and Talkington, 2004).

The attachment of MP to the host cell is a critical step in the pathogenesis, with the transmembrane adhesion protein P1 playing a key role in this process. The genome of MP contains only one copy of the functional P1 gene. The gene in question consists of two repetitive regions: RepMP4, located at the 5’ end of the coding region, and RepMP2/3, located at the 3’ end of the coding region (Spuesens et al., 2009; Waites and Talkington, 2004). Based on the P1 gene sequence, two major types (1 and 2) have been identified. The M129 strain of MP is the reference strain of type 1, while the FH or Mac are classified as type 2 (Dorigo-Zetsma et al., 2001). Various studies indicate that one of the two types typically predominates among clinical isolates in specific geographical regions, and that the predominant type can change over time. Thus, emerging changes in the P1 adhesin type may be associated with the time of disease outbreak occurrence (Waites and Talkington, 2004).

Three classes of antibiotics have been shown to be effective against MP: macrolides, fluoroquinolones, and tetracyclines (Oishi and Ouchi, 2022; Wang et al., 2024). It is well known that macrolide antibiotics, such as azithromycin and clarithromycin, are preferred for the treatment of mycoplasma infections in children due to their high efficacy and reduced toxicity in pediatric patients. The widespread usage of macrolides has inevitably led to the emergence of resistant strains, such as macrolide-resistant *M. pneumoniae* (MRMP) (Kim et al., 2022; Wang et al., 2023). Point mutations in the V domain of the 23S rRNA, especially A2063G and A2064G, which are associated with a high rate of resistance, are still the most significant. Additional (rarer) mutations include A2067G and C2617G. However, these mutations have been shown to induce a lower level of resistance compared to mutations at positions 2063 and 2064 (Bébéar et al., 2011; Bébéar and Pereyre, 2005; Oishi and Ouchi, 2022; Principi and Esposito, 2013). The resistance of MP strains to quinolones can be attributed to mutations in genes such as *gyrA*, *gyrB*, *parC*, and *parE* (Yoshida et al., 1990; Gautier-Bouchardon et al., 2002; Bébéar et al., 2003; Gruson et al., 2005; Sulyok et al., 2017). To date, there have been no clinical strains of MP that are resistant to fluoroquinolones (Wang et al., 2024). This resistance has only been observed *in vitro* (Gruson et al., 2005). However, mutations associated with resistance to fluoroquinolones have been identified in other *Mycoplasma* spp. strains (Gautier-Bouchardon et al., 2002; Sulyok et al., 2017).

Accurate diagnosis of MP is critical for clinical management, particularly given the rising incidence of macrolide-resistant strains. Current clinical diagnostics primarily rely on molecular serological assays, such as ELISA, and nucleic acid amplification (polymerase chain reaction, PCR). For the surveillance of antibiotic resistance, targeted sequencing of the 23S rRNA V domain – most commonly via PCR amplification and Sanger sequencing – remains the standard method, although high-throughput sequencing (HTS) is increasingly used for broader genomic analysis of resistance determinants (Carrim et al., 2018; Xu et al., 2024).

Thus, the importance of implementing comprehensive genomic monitoring for MP and associated viral and bacterial co-infections is driven by growing problem of MP antibiotic resistance, requiring high-precision molecular epidemiology, the negative impact of co-infections on the severity and outcomes of MPP, and limitations of current methods for MP genotyping.

The aim of the current study was to conduct molecular genetic surveillance of an outbreak of pneumonia caused by *M. pneumoniae* in various regions of the Russian Federation between January 2024 and June 2025. We developed a panel of primers to amplify the complete genes of P1 adhesin gene (*mgpA*), 23S rRNA, *gyrA*, *gyrB*, *parE*, *parC* in order to type *Mycoplasma pneumonia* according to the P1 gene and analyze mutations associated with antibiotic resistance to macrolides and fluoroquinolones. We also assessed the spectrum of viral and bacterial co-infections in all samples. Additionally, a subset of serum samples was analyzed by ELISA to support the diagnostic findings (Pereyre et al., 2016).

## 2 Materials and methods

### 2.1 Sample collection

The study was carried in the laboratory of multiomics research of the Research Institute for System biology and medicine and included 482 nasopharyngeal swabs and 75 blood serum samples obtained between January 2024 and June 2025 from patients diagnosed with pneumonia caused by *M. pneumoniae* (MPP). The inclusion criteria were as follows: patients of all ages; clinical symptoms of pneumoniae (fever, cough, and pulmonary rales) confirmed by chest X-ray, and a positive PCR test for *M. pneumoniae*.

In total, 426 children (under the age of 18) with an average age of 10.67 years (± 3.93), and 56 adults (over the age of 18) with an average age of 33.81 years (± 16.4) were included in the study.

**Figure 1:**
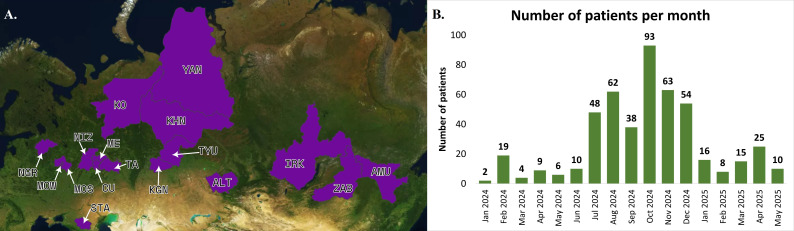
**(A)** The regions where samples were collected are marked in purple. **(B)** The number of samples obtained from patients with MPP per month.

Thus, nasopharyngeal swabs were obtained from 17 regions of Russia: the Altai region (n=10), Amur region (n=25), Transbaikal region (n=6), Irkutsk Region (n=13), Kurgan region (n=2), Moscow (n=17), Moscow region (n=6), Nizhny Novgorod region (n=134), Novgorod region (n=62), Komi republic (n=7), Mari El Republic (n=29), Republic of Tatarstan (n=15), Republic of Chuvashia (n=103), Stavropol region (n=18), Tyumen Region (n=11), Khanty-Mansi Autonomous region (n=7), Yamalo-Nenets Autonomous region (n=17) (**Figures 1A, B**).

In regional hospitals, all swabs were initially tested for MP using real-time PCR using *Mycoplasma pneumoniae/Chlamydophila pneumoniae*-FEP kits (AmpliSens, Russia) and AmpliPrime *M. pneumoniae/C. pneumoniae/S. pneumoniae/H. influenzae* (Nextbio, Russia) commercial kits.

After the primary diagnostics by PCR nasopharyngeal swabs and blood serum were collected and transported to the central reference laboratory at -20 °C, and stored at -80 °C until further processing.

### 2.2 Nucleic acid extraction, real-time PCR for *M. pneumoniae*, viral and bacterial co-infections

DNA was extracted using a Kingfisher Apex workstation (Thermo Fisher Scientific, USA) with the MagnoPrime FAST-R kit (Nextbio, Russia). The presence of MP was confirmed by real-time PCR using the AmpliPrime *M. pneumoniae*/*C. pneumoniae*/*S. pneumoniae*/*H. influenzae* kit (Nextbio, Russia). Viral co-infections were evaluated using the AmpliPrime ARVI-complex kit (Nextbio, Russia), and AmpliPrime SARS-CoV-2/Flu (A/B/H1pdm09) kit (Nextbio, Russia).

### 2.3 Primer design of the complete P1 adhesin gene (*mgpA)*

To amplify the complete sequence of the P1 adhesin gene (*mgpA*), a primer design strategy based on conserved regions was employed. Full-length *mgpA* gene sequences were extracted from all available complete MP genomes in GenBank (accession numbers provided in **Supplementary Table 2**). These sequences were aligned using MAFFT (v7.526) to identify regions of high sequence conservation. Extended regions of identity (≥100 bp) within the alignment were selected as candidate sites for primer binding. Primer pairs were designed from these conserved regions using the BacBrowser Find Primers tool (Semashko et al., 2025), with parameters set to generate amplicons ranging from 800 to 1500 bp and a melting temperature (Tm) between 58 °C and 62 °C. Candidate primer pairs were subsequently evaluated for specificity. Each pair was verified in silico to ensure it would not amplify pseudogene sequences in the M129-B7 reference genome (NC_020076). Potential for dimer formation was assessed using the Multiple Primer Analyzer tool (Thermo Fisher Scientific, USA). This process yielded two pools of primer pairs (three pairs per pool), designed to generate overlapping amplicons spanning the *mgpA* coding sequence, accounting for the known sequence variations between the P1 and P2 types of MP.

### 2.4 Primer design, amplification, and sequencing of the *23S, parC, parE, gyrA*, and *gyrB* genes

To select primers for the 23S, *parC, parE, gyrA*, and *gyrB* regions, we used the sequence of the reference strain M129-B7 reference genome (NC_020076) obtained from the GenBank NCBI. The target regions (23S rRNA, *parC, parE, gyrA, gyrB*) were chosen in accordance with published data (Chernova et al., 2016). Primers for all target regions were designed using PrimalScheme 3 (https://primalscheme.com/) and their specificity was validated in silico using Primer-BLAST (Ye et al., 2012) and the UCSC In-Silico PCR online tool (Perez et al., 2025). The primer sequences were then manually optimized and grouped into two multiplex pools (D and E). Finally, the potential for primer dimer formation within each pool was assessed using the Multiple Primer Analyzer (Thermo Fisher Scientific, USA) (**Supplementary Table 1**, **Supplementary Figure 1**).

### 2.5 Amplification with selected primers for the complete *mgpA*, 23S, *parC*, *parE*, *gyrA*, *gyrB* genes

Amplification with selected primers was performed for all 482 DNA samples. The reaction mix consisted of 7.5 μl of Q5 Hot Start High-Fidelity 2X Master Mix (New England Biolabs, USA), 2 μl of each primer pool with the final concentration of 5pM, 5 μl of sample, and 0.5 μl of H2O. The amplification program included following steps: initial preheating at 98 °C for 1 min; 35 cycles of 10 s at 98 °C, 30 s at 62 °C (for pools A and C) and 30 s at 65 °C (for pools D and E), 60 s at 72 °C; and final extension for 5 min at 72 °C. The expected amplicon sizes were approximately 950 bp for the 23S rRNA gene, 1207 bp for the *parC-parE* and *gyrB-gyrA* fragments, and two distinct products of 900 bp and 1200 bp for the *mgpA* gene. The amplification products were visualized by gel electrophoresis with a 1.5% agarose gel and the following parameters: 30 min, 180 V, 120 mA, and 25 W.

### 2.6 Sequencing of amplification products on ONT

A total of 345 samples yielding PCR products of the expected size of the target genes (23S rRNA, *parC, parE, gyrA, gyrB*, and P1) were selected for HTS. For each sample, the amplification products were then mixed and purified using magnetic beads in 1:1.2 ratio. Amplicon libraries were prepared using the Rapid Barcoding kit V14 SQK-RBK114.96 (Oxford Nanopore Technologies, UK) according to the protocol for amplicon sequencing from DNA. Sequencing was performed on the GridION and PromethION platforms (Oxford Nanopore Technologies, United Kingdom).

### 2.7 Sequencing data analysis

All sequence analyses, including in silico examination and variant calling, have been standardized using the M129-B7 (NC_020076) reference genome. The reads were processed using an in-house Nextflow pipeline, which included quality filtering and variant calling. In brief, the reads were initially processed using the Chopper (v0.8.0) and Kraken2 (minikraken2 database, v2.1.5). Reads belonging to *M. pneumoniae* were extracted and mapped to reference genes using the minimap2 program (v2.29-r1283) (Li, 2018). Variant calling was performed using Clair3 (v1.0.11), and single nucleotide polymorphism (SNP) annotation was performed using SnpEff (v5.1d).

### 2.8 Phylogenetic analysis of the complete P1 (*mgpA*) gene

For phylogenetic analysis, we used 249 complete *mgpA* gene sequences and 88 complete genomes of *M. pneumoniae* obtained from the NCBI RefSeq database, including two reference genomes (M129-B7 [NC_020076] and FH [GCF_001272835.1]). Nucleotide sequences with associated metadata were downloaded using the NCBI Datasets package (v.16.27.1). Multiple alignment of nucleotide sequences was performed using the MAFFT program (v.7.525) with an open gap penalty value of 1.53. The phylogenetic tree was constructed using the IQ-TREE2 program (v.2.0.7) with the following parameters: optimal evolution model (HKY+F+I), bootstrap support 1000 replicates. The consensus tree was then visualized using the iTOL web service (v.7.2.1).

### 2.9 Statistics

Statistical analysis was performed using Python 3.12 (pandas 2.3.3, SciPy 1.15.3). Regional differences in antimicrobial resistance rates as well as associations between co-infections and age groups were assessed using a two-tailed Fisher’s exact test with Bonferroni correction for multiple comparisons. The correlation between viral/bacterial co-infections and *M. pneumoniae* Ct values was evaluated by calculating Pearson’s correlation coefficient (for MP positive samples, being normally distributed). For analyses requiring a complete dataset, MP negative samples (Ct value not reached within 45 cycles) were assigned the value of Ct = 45, a conservative approach that preserves all patients. Spearman’s rank correlation coefficient was calculated for non-normally distributed data.

### 2.10 ELISA testing of blood serum

For a total of 75 patients from nine different regions, whose blood serum were collected, an ELISA test was performed to detect the presence of IgM and IgG antibodies to *M. pneumoniae* using the “ELISA Myco-pneum-IgM” and “ELISA Myco-pneum-IgG” kits (Ecolab, Russia). The serum samples were collected from the following nine regions: the Republic of Tatarstan (n=7), Altai Territory (n=10), Moscow (n=11), Irkutsk region (n=6), Kurgan region (n=2), Moscow region (n=6), Tyumen region (n=10), Trans-Baikal region (n=6), Yamal-Nenets Autonomous Area (n=17).

## 3 Results

### 3.1 Real-time PCR for the presence *of M. pneumoniae* and bacterial co-infections

A total of 482 nasopharyngeal swabs were obtained from patients with *Mycoplasma pneumoniae* pneumonia (MPP) hospitalized between January 2024 and June 2025. Real-time PCR confirmed the presence of *MP* in 393 samples, yielding a positivity rate of 81.5%. The largest single geographic contribution was from Nizhny Novgorod (134/482, 27.8%).

**Figure 2:**
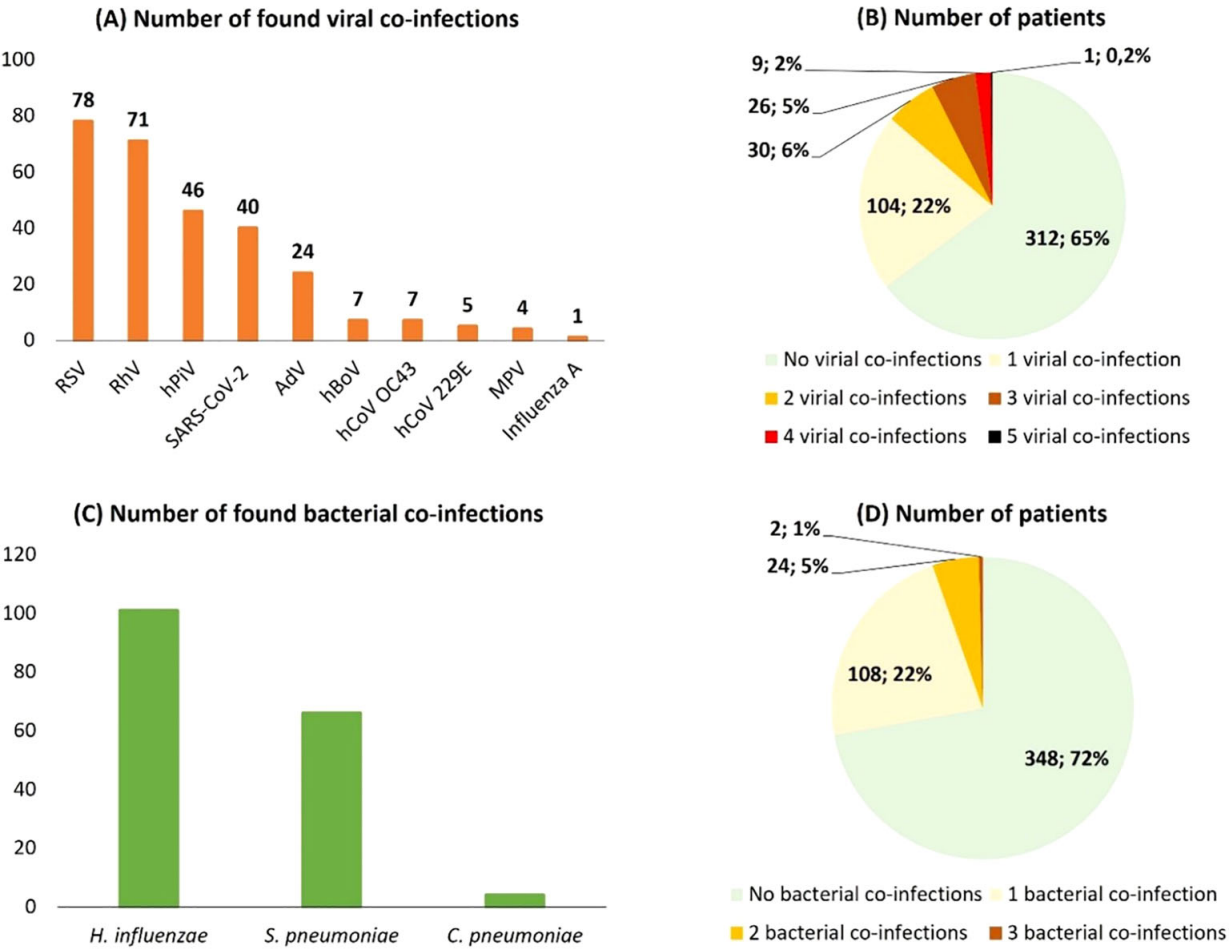
**(A)** Distribution of viruses in the studied samples; **(B)** The number of patients with one, two and three viral co-infections **(C)** Distribution of bacterial co-infections **(D)** The number of patients with one, two, and three bacterial co-infections.

All 482 samples were screened for bacterial co-infections. Overall, bacterial co-infections were identified in 134 samples (27.8%). Of these, 108 (80.6% of co-infected samples; 22.4% of all samples) had a single bacterial co-infection (mono-infection), 24 (17.9%) had two, and 2 (1.5%) had three bacterial co-infections (**Figure 2D**). *H. influenzae* was the most prevalent, detected in 95 samples (19.7%), followed by *S. pneumoniae* in 63 (13.1%) and *C. pneumoniae* in 4 (0.8%) (**Figure 2C**).

The prevalence of bacterial co-infections varied significantly across the regions. *H. influenzae* was the most frequently detected in samples from the Republic of Mari El (13/29; 44.8%), the Komi Republic (3/7; 42.9%), and the Stavropol region (6/18; 33.3%). *S. pneumoniae* and *C. pneumoniae* co-infections were the most common in Irkutsk region (38.5% and 7.7%, respectively) and Trans-Baikal region (33.3% for *S. pneumoniae*). Notably, triple bacterial co-infections were identified in samples from Irkutsk, Nizhny Novgorod, and Novgorod region. A consistent trend of co-circulation involving at least *H. influenzae* and *S. pneumoniae* was evident across all regions, with the exception of Moscow, Moscow Oblast, Altai region, and Kurgan region, where no bacterial co-infections were detected. The complete distribution of bacterial co-infections by regions provided in **Table 1**.

**Table 1:** Distribution of bacterial co-infections detected by real-time PCR.

The prevalence of respiratory bacterial co-infections (N_x_/N_all_; %)					
Region	Number of patients	*M. pneumoniae*	*H. influenzae*	*S. pneumoniae*	*C. pneumoniae*
Altai Territory	10	6/10; 60%	3/10; 30%	0	0
Amur region	25	24/25; 96%	6/25; 24%	1/25; 4%	0
Trans-Baikal Territory	6	2/6; 33,3%	2/6; 33,3%	2/6; 33,3%	0
Irkutsk Region	13	13/13; 100%	4/13; 30,8%	5/13; 38,5%	1/13; 7,7%
Kurgan Region	2	2/2; 100%	0	0	0
Moscow	17	6/17; 35,3%	0	0	0
Moscow Region	6	6/6; 100%	0	0	0
Nizhny Novgorod Region	134	111/134; 84,3%	22/134; 16,4%	18/134; 13,4%	2/134; 1,5%
Novgorod Region	62	50/62; 80,6%	13/62; 21%	8/62; 12,9%	1/62; 1,6%
Komi Republic	7	4/7; 57,1%	3/7; 42,9%	1/7; 14,3%	0
Republic of Mari El	29	29/29; 100%	13/29; 44,8%	8/29; 27,6%	0
Republic of Tatarstan	15	12/15; 80%	1/15; 6,7%	2/15; 13,3%	0
Chuvash Republic	103	81/103; 78,6%	18/103; 17,5%	16/103; 15,5%	0
Stavropol Territory	18	17/18; 94,4%	6/18; 33,3%	2/18; 11,1%	0
Tyumen Region	11	10/11; 90,9%	3/11; 27,3%	0	0
Khanty-Mansi Autonomous Area	7	5/7; 71,4%	1/7; 14,3%	0	0
Yamal-Nenets Autonomous Area	17	13/17; 76,5%	0	0	0
Summary	482	393/482; 81,5%	95/482; 19,7%	63/482; 13,1%	4/482; 0,8%

### 3.2 Analysis of the spectrum of viral co-infections

A total of 482 samples were screened by real-time PCR with the commercial panel which included respiratory syncytial virus (RSV), rhinovirus (RV), parainfluenza virus types 1-4 (hPIVs 1-4), SARS-CoV-2, human coronaviruses (229E, NL63, OC43, HKU1), adenovirus (AdV), bocavirus (hBoV), metapneumovirus (MPV), and influenza A and B viruses.

Viral co-infections were identified in 170 samples (35.3%). The most common viral co-infections were RSV (78/482 samples; 16.2%), rhinovirus (71/482 samples; 14.7%), parainfluenza viruses (46/482 samples; 9.5%), SARS-CoV-2 (40/482 samples; 8.3%), and adenovirus (24/482 samples; 5%). One virus was detected in 104 samples, two viruses in 30, three viruses in 26 samples, four viruses in 9 samples, and five viruses in a single sample (**Figures 2A, B**).

A significant prevalence of RSV (23/25 samples, 92%) as well as RV (20/25 samples, 80%) and hPIVs 1-4 (11/25 samples; 44%) was observed in the Amur region. RV (5/7 samples, 71.4%) was most frequently detected in the Komi Republic. For patients from Moscow, SARS-CoV-2 was the most prevalent (3/6, 50%). The high rate of RSV, RV and hPIVs among patients from the Amur Region might be associated with a recent nosocomial outbreak of these infections, as the samples were collected during a specific time period. The detailed information on viral co-infections is presented in **Table 2**.

**Table 2:** Distribution of viral co-infections detected by real-time PCR.

The prevalence of respiratory viral infections (N_x_/N_all_; %)											
Region	Number of patients	RSV	RV	hPIVs 1-4	SARS-CoV-2	AdV	hBoV	hCoV OC43	hCoV 229Е	MPV	Influenza A
Altai Territory	10	2/10; 20%	4/10; 40%	2/10; 20%	2/10; 20%	0	0	0	0	1/10; 10%	0
Amur region	25	23/25; 92%	20/25; 80%	11/25; 44%	0	0	2/25; 8%	2/25; 8%	3/25; 12%	1/25; 4%	0
Trans-Baikal Territory	6	2/6; 33,3%	3/6; 50%	1/6; 16,7%	1/6; 16,7%	0	0	0	0	0	0
Irkutsk Region	13	0	1/13; 7,7%	0	3/13; 23,1%	1/13; 7,7%	0	0	0	1/13; 7,7%	0
Kurgan Region	2	0	0	0	0	0	0	0	1/2; 50%	0	0
Moscow	17	5/17; 29,4%	7/17; 41,2%	3/17; 17,6%	2/17; 11,8%	0	0	0	1/17; 5,9%	0	0
Moscow Region	6	2/6; 33,3%	3/6; 50%	1/6; 16,7%	3/6; 50%	0	1/6; 16,7%	0	0	0	0
Nizhny Novgorod Region	134	23/134; 17,2%	17/134; 12,7%	13/134; 9,7%	6/134; 4,5%	2/134; 1,5%	2/134; 1,5%	2/134; 1,5%	0	0	0
Novgorod Region	62	1/62; 1,6%	2/62; 3,2%	0	3/62; 4,8%	4/62; 6,5%	0	0	0	1/62; 1,6%	0
Komi Republic	7	4/7; 57,1%	5/7; 71,4%	3/7; 42,9%	1/7; 14,3%	0	0	1/7; 14,3%	0	0	0
Republic of Mari El	29	1/29; 3,4%	0	3/29; 10,3%	3/29; 10,3%	1/29; 3,4%	0	0	0	0	0
Republic of Tatarstan	15	1/15; 6,7%	0	0	1/15; 6,7%	0	0	0	0	0	0
Chuvash Republic	103	3/103; 2,9%	1/103; 1%	0	5/103; 4,9%	14/103; 13,6%	1/103; 1%	0	0	0	0
Stavropol Territory	18	8/18; 44,4%	6/18; 33,3%	8/18; 44,4%	3/18; 16,7%	1/18; 5,6%	0	2/18; 11,1%	0	0	0
Tyumen Region	11	2/11; 18,2%	2/11; 18,2%	1/11; 9%	3/11; 27,3%	0	1/11; 9%	0	0	0	1/11; 9%
Khanty-Mansi Autonomous Area	7	0	0	0	0	0	0	0	0	0	0
Yamal-Nenets Autonomous Area	17	1/17; 5,9%	0	0	4/17; 23,5%	1/17; 5,9%	0	0	0	0	0
Summary	482	78/482; 16,2%	71/482; 14,7%	46/482; 9,5%	40/482; 8,3%	24/482; 5%	7/482; 1,5%	7/482; 1,5%	5/482; 1%	4/482; 0,8%	1/482; 0,2%

### 3.3 Statistical analysis

As mentioned above, among the 482 patients, 393 were positive for MP (Ct < 45). Ct values were approximately normally distributed (Shapiro–Wilk, statistics=0.996, p-value=0.349; **Supplementary Figure 3**). For analyses requiring a complete dataset, undetected samples (Ct not reached within 45 cycles) were imputed to Ct = 45, a conservative approach that preserves all patients.

A statistically significant negative monotonic relationship was observed between MP Ct and the number of bacterial co−infections. The effect size was small (~-0.2), indicating that a higher bacterial co−infection count is associated with higher MP load. This finding was robust in a sensitivity analysis restricted to patients with detectable MP. Using Pearson’s correlation (appropriate given the normality of Ct in this subset), the association remained significant and negative, with a similar small effect size.

No statistically significant association was detected between *M. pneumoniae* Ct and the number of viral co−infections in either analysis.

For statistical analysis of the regional differences in macrolide resistance rate six regions with more than 15 samples were included: Amur region (n=24), Nizhny Novgorod Region (n=77), Novgorod Region (n=31), Republic of Mari El (n=27), Chuvash Republic (n=68) and Stavropol Territory (n=17). A two-tailed Fisher’s exact test with Bonferroni correction (p=0.008) revealed no significant differences in resistance rates among these regions or when compared to all studied regions (**Supplementary Table 5.1**).

Associations between co-infections and age were assessed by categorizing patients into three groups: preschool (0–6 years old, n=73), school-age children (7–17 years old, n=367), and adult (>18 years, n=62). The two-tailed Fisher’s exact test with a Bonferroni correction (p=0.003) showed no significant difference in the prevalence of either viral or bacterial co-infections among these age groups (**Supplementary Table 5.2**).

### 3.4 Development of the primer panel for amplification of the complete *mgpA, 23S, parC, parE, gyrA, gyrB* genes and analysis of the identified mutations

To analyze mutations in the genes responsible for resistance to macrolides and fluoroquinolones, and to perform genotyping based on the P1 adhesion protein gene, we designed primers for amplifying the complete sequences of the *23S rRNA*, *parC*, *parE*, *gyrA*, *gyrB*, and *mgpA* genes. In total 18 primers were selected and combined into four multiplex pools. Pools A and C each contained three primer pairs for amplifying the complete *mgpА* gene, while Pools D and E each contained six primer pairs for amplifying the complete *23S rRNA*, *parC*, *parE*, *gyrA*, and *gyrB* genes (two primer pairs per gene).

Following purification with magnetic beads, the resulting PCR products with sizes of 900 bp and 1200 bp were taken for sequencing on the Oxford Nanopore Technologies platform. The primer sequences and an example of amplification result using DNA from clinical samples from patients with MPР provided in **Supplementary Table 1**, **Supplementary Figure 2**, respectively.

Amplification with the designed primer pools was performed for all 482 DNA samples. PCR products were detected in samples with a Ct value below 38. Consequently, 342 samples were selected for sequencing and subsequent analysis.

### 3.5 Genotyping of *Mycoplasma pneumoniae* based on phylogenetic analysis of the complete P1 gene (*mgpA*)

**Figure 3:**
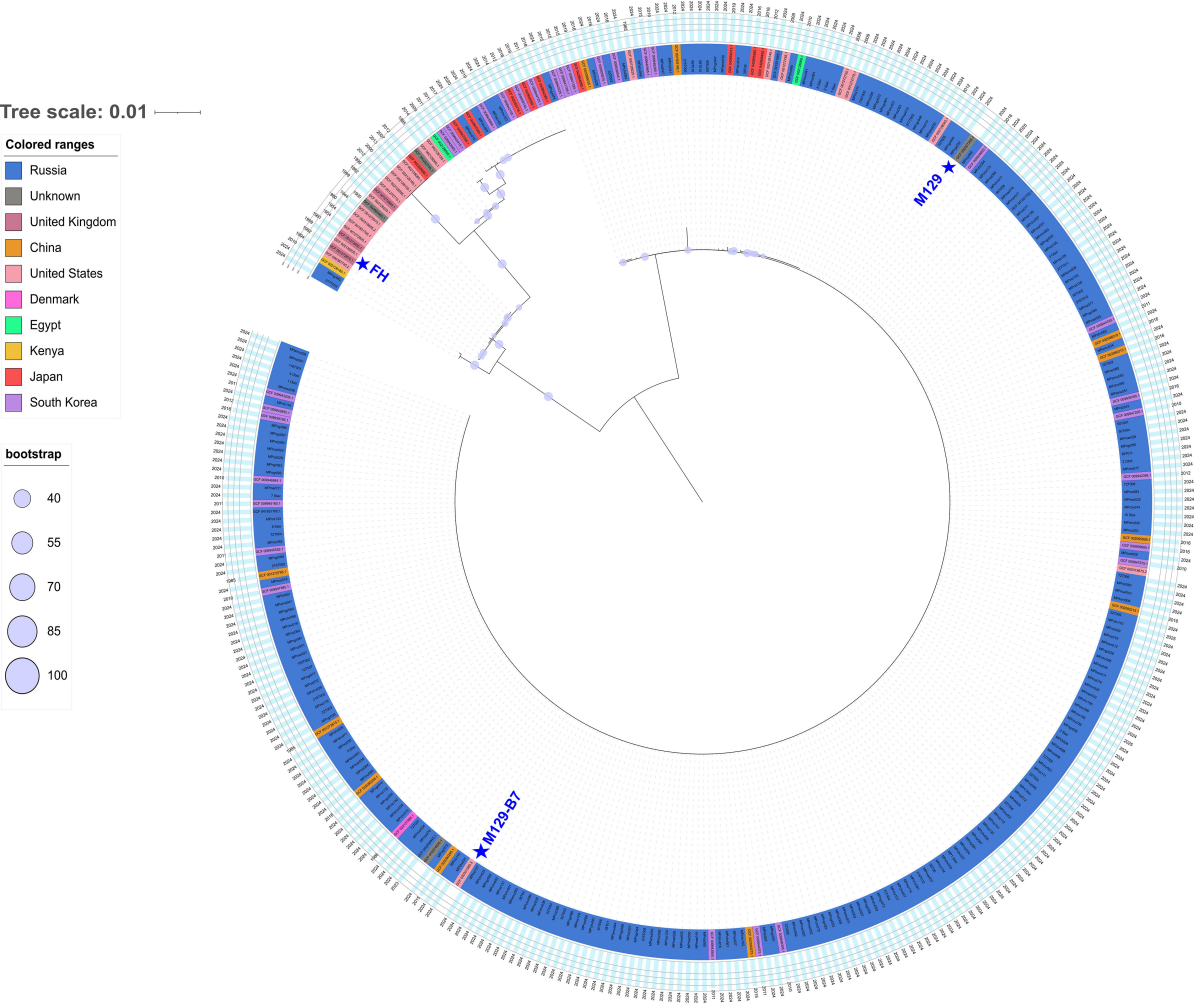
A maximum likelihood-based phylogenetic tree constructed for 249 samples and reference strains (M129-B7 [NC_020076] and FH [NZ_CP010546.1]) with the bootstrap value 1000. Sequences originating from Russia are marked in blue.

As described above, amplification products of the complete *mgpA* gene were obtained for 342 samples, which were subsequently sequenced on the Oxford Nanopore Technologies platform. Following quality filtering, 249 high-quality sequences were taken to phylogenetic tree construction. The analysis revealed that the *mgpA* gene sequences formed two distinct clades. Of the 249 sequences, 242 (97.2%) were classified as type 1, clustering with the reference strain M129-B7 (NC_020076) – P1 type 1, while the remaining 7 (2.8%) were classified as type 2 and clustered with strain FH (NZ_CP010546.1) – P1 type 2 (**Figure 3**).

The P1 type 1 clade included the sequences from China and South Korea, with additional representatives from Denmark, the USA, and Russia. In contrast, the P1 type 2 clade is primarily comprised of sequences originating from the USA, the UK, and China.

### 3.6 Analysis of mutations in the V region of 23S rRNA associated with resistance to macrolides

23S rRNA gene sequences were analyzed in 342 samples. The sequences were analyzed against the reference genome M129-B7 (NC_020076). For isolates identified as P1 type 2, a parallel analysis was conducted using known differences between the FH and M129-B7 reference genomes to ensure accurate variant calling in type-specific regions.

Nucleotide substitutions were identified in 90 samples (26.3% of 342). The spectrum of mutations included known resistance-associated variants and substitutions of unknown significance (**Supplementary Table 3**).

The primary mutation associated with macrolide resistance, A2063G, was assessable in 335 samples and was detected in 77, yielding a prevalence of 23.0% (77/335). Another point mutations located in 2064 position associated with macrolide resistance to 14- and 15-membered macrolides was found in 6 samples (A2064G – 3 samples, A2064C – 3 samples). The highest prevalence of these resistance mutations was observed in samples from the Altai region, Kurgan Region, and Moscow.

Additionally, we detected the C1620G substitution in two P1 type 1 samples (2 out of 337 with evaluable sequence data). To our knowledge, the C1620G variant has not been previously associated with macrolide resistance (**Supplementary Table 3**).

#### 3.6.1 Analysis of resistance to fluoroquinolones in the *gyrA, gyrB, parC*, and *parE* genes

Mutations associated with fluoroquinolone resistance were analyzed in 342 samples, and nucleotide substitutions were identified in 314 (91.8%).

In the *gyrA* gene synonymous mutations were highly prevalent, most notably G369T (97.7%). Other synonymous substitutions were also identified, A2475G (1.8%), C1023T (0.3%), G1194A (0.3%). Non-synonymous mutations in *gyrA* A446G (p.Asp149Gly) was revealed in 5 samples.

In the *gyrB* gene non-synonymous mutations were detected at low frequencies: C262T (p.Pro88Ser; 0.3%), T1094C (p.Leu365Pro; 0.3%), T1181C (p.Leu394Ser; 0.3%), G1264A (p.Gly422Ser; 0.6%);, T1814C (0.3%; p.Val605Ala), while the synonymous substitutions identified was C1644T (0.3%).

Analysis of the *parC* gene revealed the synonymous mutation A900G in all samples analyzed. The non-synonymous substitutions identified were A637G (p.Lys213Glu, 0.7%).

In the *parE* gene, we identified three synonymous mutations: C255A (0.3%), G447A (1.7%), G462T (0.7%) (**Supplementary Table 3**).

However, the identified substitutions are located outside the canonical QRDR, and their contribution to fluoroquinolone resistance remains unclear.

### 3.7 Determination of IgM and IgG antibodies to *M. pneumoniae.*

Serological analysis of 75 patients from nine regions was performed by ELISA to detect *M. pneumoniae*-specific IgM and IgG antibodies. Overall, 69.3% (52/75) of patients were seropositive (positive for either IgM or IgG). Specifically, anti-*M. pneumoniae* IgM was positive in 57.3% (43/75) of patients, while IgG was detected in 30.7% (23/75). A notable finding was observed in the 21 patients (28.0%) with negative RT-PCR results: among them, 14 were seropositive, with profiles ranging from IgM-only (n=8) to IgG-only (n=1) and dual-positive (n=5) (**Supplementary Table 3**).

## 4 Discussion

This study presents the results of surveillance for MPP in patients from 17 regions of the Russian Federation between January 2024 and June 2025. The study cohort consisted of 426 children and 56 adults, with a mean age of 13 years (range: 0–78 years). While MPP is predominantly considered as a childhood disease, our cohort included a significant number of adults, including patients over 65, underscoring its broader clinical relevance. Furthermore, we observed a fundamental shift in the epidemiology of MPP. Contrary to the classic 3-7-year epidemic cycles, our data reveal endemic, year-round transmission since 2023, characterized by a sustained peak from the middle of the summer to the winter. This shift towards endemic transmission may be attributed to an increased susceptibility to respiratory infections in the population following the COVID-19 pandemic, leading to more frequent detection of MP (Meyer SauteurEuropean Society of Clinical Microbiology and Infectious Diseases Study Group for Mycoplasma and Chlamydia Infections (ESGMAC) Mycoplasma pneumoniae Surveillance (MAPS) study group, 2025).

Given that MP was identified as both a primary pathogen causing MPP and a co-infection in immunocompromised hosts, we analyzed patient samples for the presence of bacterial co-infections commonly associated with CAP, including *H. influenzae, S. pneumoniae*, and *C. pneumoniae*. Bacterial co-infections were detected across all studied regions except Moscow, Moscow Region, and Kurgan Region. Globally, the bacterial co-infections with MP is similar, though detection rates vary. The most frequently identified co-infections in patients with CAP worldwide are *S. pneumoniae, H. influenzae*, and *S. aureus* (Liu et al., 2025). in China, *S. pneumoniae* and *H. Influenzae* are reported as the dominant bacterial co-infection in children with CAP (Yuan et al., 2025; Zhang et al., 2018). In Europe, similar patterns are observed, where *S. pneumoniae* and *H. influenzae* are the most frequently identified bacterial co-infections during MP infection (Waldeck et al., 2025).

Among viral co-infections, respiratory syncytial virus (16.2%), rhinovirus (14.7%), and parainfluenza viruses (9.5%) were the most prevalent, followed by SARS-CoV-2 (8.3%) and adenovirus (5.0%). This represents a shift from our previous study (January 2023–February 2024), where human parainfluenza viruses (hPIVs 1-4) and SARS-CoV-2 were the dominant co-infecting pathogens (Korneenko et al., 2025). The most common viral co-infections with *M. pneumoniae* are similar across China, the USA, and Europe, primarily, including rhinovirus (Yuan et al., 2025), adenovirus (Chen Q. et al., 2024; Li et al., 2022), RSV, Influenza viruses (Yu et al., 2024) and SARS-CoV-2 (Li et al., 2024; Chi et al., 2024; Zhao et al., 2025).

The distribution of viral and bacterial co-infections does not demonstrate any general patterns but rather is associated with the prevalence of the circulating pathogens in a particular region.

In order to type the MP according to P1 adhesin sequences and to analyze mutations associated with resistance to macrolides and fluoroquinolones, a panel of primers was developed for the amplification of complete 23S rRNA, *gyrA, gyrB, parC, parE*, and *mpgA* genes. The developed method has been tested on a collection of clinical samples and is suitable for use in laboratories with platforms for long read sequencing, such as Oxford Nanopore Technologies.

Typing based on the complete *mgpA* (P1) gene sequence revealed that the majority of strains belonged to type 1, while seven sequences were classified as type 2. Although the absence of clinical data precludes correlation with disease presentation, the identification of this type 2 strain suggests a potentially distinct importation event, likely from a different geographic origin.

The designed panel allows obtaining information on nucleotide substitutions in genes associated with resistance to fluoroquinolones and macrolides. The frequency of the A2063G mutation in the 23S rRNA gene was 23%, which is lower than the level of resistance observed in the period from January 2023 to February 2024 (Korneenko et al., 2025). We observed geographic heterogeneity in macrolide resistance rates, ranging from 0% to 50%. The highest rates were found in Moscow, the Moscow region, the Kurgan region, and the Altai region, whereas the lowest rates were detected in the Trans-Baikal region, the Irkutsk region, and the Republic of Tatarstan. The mutation at position 2064 (A2064G, A2064C), which is less prevalent but also associated with macrolide resistance, has been detected in isolated samples from patients in the Nizhny Novgorod region, the Republic of Tatarstan, and Chuvashia. These three regions are in close proximity, and the spread of A2064G may be associated with geographical location. The global incidence of MRMP in 2023 and 2024 still varies significantly, with rates remaining low in the United States (7,1%) (Edens et al., 2024), Europe (1-25%) (Varela et al., 2023), and Japan (14.3%) (Nakamura et al., 2021), while reaching extremely high levels in China (99%) (Chen Y. et al., 2024; Xu et al., 2024).

Comprehensive sequencing of the *gyrA, gyrB, parC*, and *parE* genes from 342 clinical samples revealed a high prevalence of nucleotide substitutions. The identification of recurrent non-synonymous mutations, such as *gyrA* Arg792Gly, located outside the canonical QRDR, suggests that these sequence variations represent natural polymorphisms rather than acquired mechanisms of fluoroquinolone resistance (De Groot et al., 2025). Further analysis is required to determine the significance of these polymorphisms.

The statistical analysis revealed no significant epidemiological associations. Specifically, we found no correlation between geographic region and macrolide resistance rates, no association between patient age and co-infection status, and no significant temporal trends were observed across months or seasons. However, it should be noted that a weak inverse relationship between MP Ct-value and the number of bacterial co-infections was observed. Notably, this relationship was specific to bacterial pathogens, as no association was found for viral co-infections.

Thus, in light of global data and our own observations, questions arise regarding changes in the course of MP disease following the COVID-19 pandemic. Notably, in the post-pandemic period, patients with CAP have demonstrated more severe disease, including higher fever exceeding 38 °C (**Supplementary Table 4**). Some primary factors may explain this phenomenon. First, the “immunity debt” hypothesis suggests that non-pharmaceutical interventions (NPIs) reduced the circulation of MP and other respiratory pathogens for 2–3 years, leading to a large, immunologically naive cohort, particularly among young children (Boyanton et al., 2024). The subsequent lifting of restrictions resulted in an explosive outbreak within this susceptible population (Cohen et al., 2021). Second, post-COVID-19 immune dysregulation may be a contributing factor. Protracted COVID-19 can cause immune system alterations, including prolonged systemic inflammation (elevated IL-18, IL-6, IL-8, TNF-α), aberrant T-cell differentiation, and impaired B-cell maturation. These changes lead to endothelial dysfunction and increased host vulnerability to secondary infections (Boyanton et al., 2024). Supporting this, Korobova et al. demonstrated that immune profiles in healthy donors sampled after the pandemic’s onset differed from pre-pandemic samples, indicating that the pandemic’s consequences extend beyond acute infection or long COVID (Korobova et al., 2025).

Thus, we also observe that the COVID-19 pandemic disrupted the conventional epidemiology of respiratory pathogens, including *Mycoplasma pneumoniae*. The subsequent lifting of restrictions triggered an “immunity debt,” now evident as atypical, year-round outbreaks of increased severity.

The serological analysis reveals a high seroprevalence of MP infection, with 69.3% (52/75) of patients showing a positive IgM or IgG immune response. Interestingly, a notable proportion (28%) of PCR-negative samples were seropositive, underscoring the importance of integrating PCR with serological testing for comprehensive diagnosis. This approach allows for the confirmation of active infection or recent exposure through serology, even when nucleic acids are no longer detectable.

This study has some limitations. First, the negative PCR results in some seropositive samples (IgG/IgM positive) could be attributed to suboptimal sample collection, improper transportation conditions, or the inherent limitations of PCR sensitivity. Another limitation of the study includes incomplete metadata presented for the patients enrolled in the current study.

## 5 Conclusion

The results of a multicenter study conducted between January 2024 and June 2025 demonstrate an ongoing epidemic outbreak of pneumonia caused by *M. pneumoniae*, affecting patients of all ages. The present study has demonstrated that the prevalence of bacterial and viral co-infections is associated with a specific geographical area. The most prevalent viral co-infections were RSV (16.2%), rhinovirus (14.7%), and parainfluenza viruses (9.5%). The predominance of the severe acute respiratory syndrome (SARS)-coronavirus 2 (SARS-CoV-2) was observed in Moscow and less in Central Russia, while no cases were detected in patients from the Amur Region. The development of a method for amplifying the complete *23S rRNA*, *parC*, *parE*, and *gyrA*, *gyrB* genes followed by sequencing allowed us to obtain more detailed information about nucleotide substitutions in the regions associated with resistance to macrolides and fluoroquinolones. The findings of the present study demonstrate a decline in the level of resistance to macrolides to 23%, and the appearance of samples belonging to P1 type-2 clade in comparison to the autumn-winter period of 2023-2024, during which resistance was 40% and the only P1 type 1 was detected.

## Data Availability

The datasets presented in this study can be found in online repositories. The names of the repository/repositories and accession number(s) can be found in the article/**Supplementary Material**.
